# Partial privatization and intellectual capital: The moderating role of firm internationalization and regional development

**DOI:** 10.1371/journal.pone.0340906

**Published:** 2026-01-23

**Authors:** Haobo Xie

**Affiliations:** School of Continuing and Lifelong Education, National University of Singapore, Singapore; Instituto Tecnologico Autonomo de Mexico, MEXICO

## Abstract

This study examines the impact of partial privatization on intellectual capital and investigates how corporate internationalization and regional development moderate this relationship. Using a sample of Chinese listed firms over the years of 2010–2023, this study employed Ordinary Least Squares (OLS) as the baseline regression. This study finds that partial privatization positively affects intellectual capital, thereby introducing opportunities about value creation within firms. Further investigations reveal that internationalization weakens the positive impact of partial privatization on intellectual capital, suggesting that exposure to international markets may heighten agency risks associated with a firm’s global expansion. Our findings also show that regional development strengthens this positive relationship, since firms located in developed regions are better positioned to capitalize on the benefits of partial privatization. To ensure the robustness of these findings, we also use two Generalized Method of Moments (GMM) and Propensity Score Matching (PSM) methods to ensure the consistency of the findings. Overall, the results carry significant policy implications, especially concerning the corporate efficiency outcomes of privatization in emerging markets such as China.

## 1. Introduction

In China, most of SOEs are partially privatized and their shares are available to private investors but the state remains major shareholder. This allows China to attract private capital and improve efficiency while maintaining strategic oversight and influence over key industries. The privatization role in China has been thoroughly researched, particularly regarding their contributions to employment [1], industrial advancement [2], economic development [3], and green performance [4]. However, the state-owned assets are often sold at significantly undervalued prices which leads to losses for the public sector and unfair advantages for private investors [5]. Based on these contradictory views, we endeavor to dig out deeply the true outcomes of partial privatization on intellectual capital with the moderating role of internationalization and regional development which play significant role in a country’s economic growth and a firm’s competitive advantage.

Previous evidences contend that ownership structure can support firms in achieving green performance by encouraging sustainable development [6]. Likewise, Boubakri, Guedhami, Kwok, and Wang [7] explored the link between privatization and corporate social performance, while [6] investigated the relationship between different levels of state ownership and green innovation (GI). On the other hand, Yuan et al. [8] revealed how a mixed-ownership structure in state-owned enterprises (SOEs) influences environmental firm performance. However, the impact of partial privatization on intellectual capital remains largely underexplored.

The privatization processes can sensitize intellectual capital development, since it may enhance an organization’s ability to harness and optimize its unique resources—such as specialized knowledge, skills, and relationships [9,10]. The property rights promote the innovation, technology, foreign investment and financial structure of the country. In developing countries, firms have more tangible assets as compared to intangible assets but in advanced countries the firms have more proportion of intangible assets. This difference is due to weakness of property right laws and financial system. Due to poor property rights protection, the firms try to secure their return by investing more in fixed assets. The fixed assets give the certain return every year but intangible assets have the risky return [11]. Therefore, privatization can secure the investment through strict regulations and monitoring system. In such scenario, internationalization can negatively moderate the relationship, as companies entering global markets often face considerable operational and financial demands which may divert resources from intellectual capital development [12,13]. While the relationship between intellectual capital and partial privatization can be positively moderated by regional development. In particular, a regional investment in skilled people, infrastructure, and innovation-oriented setting creates a situation that stimulates innovation and intellectual capital. For example, research indicates that geographical and organizational proximity, along with regional government R&D funding, enhance innovation outcomes by providing enterprises with necessary resources and support networks [14].

The present research work considers Chinese listed firms because of its distinct institutional framework, ownership structure, and rapid shift and different attitude toward privatization programs. Mostly, Chinese state-owned enterprises depends upon governmental support to access critical resources for new product development such as patent approvals and funding for environmental initiatives [15]. Since such kind of dependency allow SOEs to get advantage from favorable regulatory environments which can accelerate product development in technology and research and development sectors [16]. However, with partial privatization, these dynamics may change by creating new motivations for SOEs to pursue independent innovation and acquire intellectual property on their own [17]. Thus, motivating from the consequences of privatization, this study explores the following question: how does partial privatization influence intellectual capital development in China?

By answering the aforementioned question, this research makes few contributions to the prior literature. For instance, prior studies have mainly concentrated on either economic impact at the macro level or financial and social performance at the micro level when analyzing privatization outcomes [7,18–20]. At the firm-level analysis, our knowledge of how secondary privatization influences intellectual capital efficiency remains in its early stages. The current research work adds insights to the literature by examining the impact of partial privatization on intellectual capital in Chinese context. Specifically, this study goes beyond to investigate the capital outcomes of partial privatization. Second, this study contributes by the examining the moderating influence of firm internationalization and regional development on the relationship between partial privatization and intellectual capital. By doing so, this study submits that regional development serves as an external monitoring mechanism that enhances the enforcement of efficient policies within privatized companies and therefore, it affects the positive link between privatization and intellectual capital efficiency. Third, by investigating how partial privatization drives intellectual capital, our work further adds that internationalization weakens the positive relationship among privatization and intellectual capital. In this sense, our research shows that the relationship between partial privatization and intellectual capital depends on the firms’ industrial setting and the degree of growth within the region and different culture. Lastly, we add to the minor part of the expanding body of research on intellectual capital by demonstrating that businesses that participate in patents or trademarks due to partial privatization perform well. When considered collectively, this research clarifies how the Chinese government’s privatization approach helps to safeguard the intellectual capital.

## 2. Related theories and hypothesis development

### 2.1. Partial privatization and intellectual capital development

Several empirical studies and related theories indicate that change of ownership in SOEs and partial privatization significantly improves firm performance in general and intellectual capital in particular [21–25]. For instance public choice theory theorizes how self-centered behaviors of public officials, politicians, and managers affect the allocation of resources within public enterprises [26,27]. This theory suggests that the misaligned incentives often lead to inefficiencies [28]. Thus, shifting a part of control to private stakeholders significantly mitigates issues stemming from political influences and bureaucratic management [29,30].

In addition, public officials managing SOEs often prioritize personal or political objectives over organizational efficiency [16], which often leads to lack of investment in human capital, innovation, and knowledge management [31,32]. For instance, they tend to avoid potentially unpopular decisions [33], such as performance based compensation or workforce optimization, either due to political pressures or a desire to maintain public favor [34]. This leads to reduced incentives for employees to develop or apply their skills innovatively [35]. According to the public choice theory, the introduction of private shareholders creates a new layer of accountability because private investors reduce the influence of political objectives and are more concerned with operational efficiencies and value creation [36,37]. This can encourage mangers to adopt policies for retention and development of intellectual capital through trainings, talent management initiatives, and knowledge-sharing systems [32,38].

Similarly, agency theory also supports the role of partial privatization in mitigating inefficiencies and the principal-agent problems [39–41]. Since, privatization helps in aligning management incentives with expectations of private stakeholders and introduces a profit motive and competitive pressure [42,43]. Consequently, firms can utilize intellectual capital more effectively as the focus is shifted towards productivity, innovation and other strategic areas that enhance the overall firm value [30,44,45]. This shift ultimately leads to improvement in knowledge management practices and human capital investment [39,46].

In addition, the perspectives of resource-based theory also favor the positive role of partial privatization in intellectual capital development because partial privatization often improves the organization’s ability to leverage its unique resources [45,47,48]. The injection of private capital in SOEs facilitates access to advanced technologies, industry expertise, and modern management practices which ultimately increases the value of its intellectual capital [48]. With these new resources, the firm can initiate training programs and improved knowledge-sharing systems to enhance employees’ skills and innovation [49,50].

Similarly, the empirical research also supports the positive relationship between partial privatization and intellectual capital development. For example, several studies on Chinese SOEs found positive effect of partial privatization on corporate innovation [5,51,52]. A study in the Iranian context by Fotros and Beygi [53] also reports a positive relationship between partial privatization and intellectual capital development. Similarly, Omran [23] also reports superior intellectual capital performance in the private sector compared to SOEs. Thus, building upon these evidences, we propose the following hypothesis:

***H1*** *: Partial privatization has a positive effect on intellectual capital development.*

### 2.2. Moderating role of Internationalization

While partial privatization plays an important role in the development of intellectual capital, their relationship is complicated by several challenges. These challenges make it difficult to utilize intellectual resources effectively. Extant literature highlights the several external factors that alter the impact of partial privatization on intellectual capital, such as misalignment of interest between private owners and internationalization. Internationalization introduces operational challenges and competitive pressures that complicate organizational management and resource allocation [54,55]. The perspectives of agency theory suggest that internationalization amplifies information asymmetry and communication challenges, particularly in the firms where private and public ownership exist together [30,56]. Moreover, managers have to allocate resources across multiple markets and comply with the diverse regulatory and cultural requirements [57]. These challenges either dilute the effectiveness of intellectual capital investment or reduce the urge for such investments [58,59].

On the contrary, the perspectives from institutional theory posit that internationalization exposes corporations to different institutional pressures that are usually not aligned with their domestic practices and priorities [60,61]. In case of partially privatized SOEs, this exposure often leads to conflict of interests between state and private interests [62]. This is because state interests often prioritize the stability of local economy, while private interests seek competitive advantage in foreign markets [63]. In the presence of such conflicts of interests, the firm may find it difficult to develop intellectual capital because instead of focusing on employee knowledge and innovation capabilities, resources are directed towards managing cross boarders risks and complying with international regulatory requirements [60,64,65].

The recent empirical studies also suggest that the relationship between partial privatization and intellectual capital development is significantly weakened by internationalization. For instance, Zhang et al. [25] and Meyer et al. [60] suggest that SOEs’ engagement in high levels of internationalization hinders the effective management of intellectual capital because cross-border complexities induce reduced emphasis on internal knowledge-sharing initiatives. Similarly, Fang et al. [22] and Chen, Xie, and Van Essen [66] found that SOEs with international operations prefer short-term performance metrics over long term human capital investment due to the pressure of global competition. These findings suggest that internationalization complicates efforts to align resources with intellectual capital development. In light of these theoretical and empirical insights, the following hypothesis is proposed:

***H2*** *: Internationalization negatively moderates the relationship between partial privatization and intellectual capital.*

### 2.3. Moderating role of regional development

Regional development also plays an important role in modifying the relationship between partial privatization and intellectual capital [67]. Regional development encompass economic growth, development of infrastructure, and the availability of skilled and capable human resource [68]. In addition, theories of regional economic development suggest that developed regions usually have robust economic ecosystems that offer conducive environment and necessary resources for optimization [69,70]. Therefore, SOEs with mixed ownership structures can perform better in developed regions [71]. Moreover, the firms in developed regions are in a better position to utilize intellectual capital due to the innovation-friendly environments [67]. Thus, regional development enables the partially privatized firms to improve their performance and achieve innovation objectives [45,72].

In similar vein, the institutional theory emphasize the role of subnational institutions and regional strategies in shaping corporate behaviors and capabilities [73]. On the basis this theory, more advanced regions have conducive institutional frameworks including the supportive legal systems, robust financial markets, collaborative networks, and established innovation ecosystems [74]. Therefore, these supportive institutional frameworks facilitate reforms and encourage knowledge sharing in SOEs [75]. Moreover, it reduces various constraints to the development and utilization of intellectual capital such as misallocation of resources and strategic misalignment [63,65]. Thus, partially privatized firms thrive well and excel in intellectual capital development in such environments.

Taken together, the prevailing evidences on significant role of regional development also provide signal that regional development could serve as monitoring factor in strengthening the relationship between partial privatization and intellectual capital. Several studies suggest that innovation and knowledge sharing is valued in the regions that have well-developed infrastructure, strong educational institutions, and vibrant business networks (See for example, [76–79]). In addition, X. Zhang et al. [25] found that SOEs with mixed ownership witness superior performance in terms of developing intellectual capital in economically advanced regions compared to less developed areas. This is because firms operating in the developed regions benefit from regional knowledge spillovers and have access to a highly skilled workforce [65]. Similarly, Yaseen [48], P. Wu and Sun [67] and Omran [23] also suggest that the effect of privatization on firm performance is more profound in the regions with supportive economic policies and innovation-friendly environments. Thus, keeping in view the above discussion the following hypothesis is proposed:

***H3*** *: Regional development positively moderates the relationship between partial privatization and intellectual capital.*

## 3. Methodology

### 3.1. Data and sample

Our sample firms initially consists of all listed firms in Shanghai and Shenzhen stock exchanges spanning the period from 2010 to 2023. We select 2010 as a starting point since data on intellectual capital (IC) factors is not well-available before 2009 and 2009 year most of the data on IC still not completed. So, to avoid such uncompleted data, we chose 2010. While 2023 is the latest year. We excluded all financial institutions as they have different financial statements. Our final sample consists of 13,809 firm-year observations. All data of our model’s variable was obtained from the Chinese Security Market Research (CSMAR) and terminal WIND databases. To eliminate the impact of outliers we use the winsoration technique at the level of 1% on both sides for all continuous variables.

### 3.2. Variables measurement

Dependent variable: Our dependent variable is Intellectual Capital (MVAIC) [80,81]. Basically, to calculate the MVAIC the model uses the components of the financial statements of a firm. However, we use three steps to calculate the MVAIC as follows.

Step one, Calculating the value added (VA) by all there sources during the year t.


$${VA}_{i,t}={OUT}_{i,t}-{IN}_{i,t}$$ (1)

where OUT is total income from in a year t, IN is all expenses (including labor expenditures) incurred by a bank for a year t.

Step two, calculating intellectual capital efficiency as follows:


$${ICE}_{i,t}={HCE}_{i,t}+{SCE}_{i,t}+{RCE}_{i,t}$$ (2)

where HCE is a proxy measures the value has been added by financial investment in employees, while SCE refers to the capital has been generated by social capital (SC), RCE is a proxy measures the value has been generated by investing one unit of RC during a year t in the bank i.


$${HCE}_{i,t}={VA}_{i,t}/{HC}_{i,t}\;$$ (3)

$${SCE}_{i,t}=({VA}_{i,t}-{HC}_{i,t}/{VA}_{i,t})$$ (4)

$${RCE}_{i,t}={RC}_{i,t}/{VA}_{i,t}$$ (5)

HC refers to the salary and wages of the employees, while RC is selling, marketing, and advertising expenditures during a year t in a bank i.

Step3, calculating the capital employees efficiency (CEE) which measured the value has been added per one USD invested by a bank i in a year t.


$${CEE}_{i,t}={VA}_{i,t}/{CE}_{i,t}$$ (6)

CE is the difference between total assets and total liabilities. Then the intellectual capital (MVAIC) can be calculate as follows:


$${MVAIC}_{i,t}={ICE}_{i,t}+{CEE}_{i,t}$$ (7)

While MVAIC is derived from financial and accounting data, which may not fully reflect all intangible knowledge assets, it remains one of the most widely used and empirically validated proxies for intellectual capital efficiency in large-scale firm-level studies. Alternative or supplementary proxies, such as patent counts, R&D expenditure intensity, or innovation output indicators, capture specific dimensions of intellectual capital (e.g., innovation capability), but they are often limited by data availability. On the other hands, along with following prior studies [80,81], MVAIC provides a comprehensive and standardized measure that reflects the combined contribution of tangible and intangible resources to firm value creation. Therefore, we adopt this proxy as a reliable and practical proxy for intellectual capital in our empirical analysis.

Independent variable: Our independent variable is Privatization According to Chen et al. [18], Khan et al. [16], and Usman et al. [4], we define this variable as reduction in state ownership by selling state ownership to private investors partially. We use three proxies for this variable (1) PRIVATEPERCE we measure this variable as a percentage of reduced share ownership by the state. Following Usman et al. [4], we also adopt the threshold approach and determine the level of privatization based on a 10% or 20% cap on state ownership. (2) PRIVATDUM10P is a dummy variable coded 1 if a firm has sold 10% or more shares from its portion of state ownership and 0 otherwise. (3) PRIVATDUM20P is a dummy variable coded 1 if a firm has sold 20% or more shares from its portion of state ownership and 0 otherwise. Theoretically, these thresholds reflect meaningful shifts in ownership concentration and control. Ownership theory proposes that investors with less than 10% equity typically lack the voting power or board representation needed to influence managerial decisions or strategic direction. In contrast, crossing the 10% or 20% thresholds represents a transition from a purely passive stake to a level that can exert significant minority influence. Thus, a sale of 10% of state ownership indicates the beginning of effective privatization, where private shareholders start influencing firm operations, while 20% and more indicates substantial privatization, potentially reducing the state’s control rights and political intervention.

Moderating variables: Since we intend to explore the implication of privatization of state ownership, we utilize the following moderators; The first is regional development, following prior studies (e.g., [82]), we measure this proxy as a dummy variable coded one if the firm is located in developed regions of China, and zero otherwise. The second is firm internationalization (INTERNATION), Second, firm internationalization (FOREIGN), following Zhang et al. [83] and measure as the proportion of foreign revenue to total sales.

### 3.3. Empirical model

To examine our hypothesis we use the following ordinary least square (OLS):


$$\begin{array}{cc} {MVC}_{i,t}= & \;\beta_{\text{0}}+\beta_{\text{1}}{PRIVATE}_{i,t}+\beta_{\text{2}}{\text{GROWTH}}_{i,t}+\beta_{\text{3}}{\text{FAGE}}_{i,t}+\beta_{\text{4}}{\text{SIZE}}_{i,t}+\beta_{\text{5}}{\text{ROA}}_{i,t}+\beta_{\text{6}}{\text{LEVERAGE}}_{i,t}\\ & +\beta_{\text{7}}{\text{TOBINQ}}_{i,t}+\beta_{\text{8}}{\text{INSTITUTION}}_{i,t}+\beta_{\text{9}}{\text{REGIOND}}_{i,t}+\beta_{\text{10}}{\text{BOARD}}_{i,t}\\ & +\beta_{\text{11}}{\text{BINDP}}_{i,t}+\beta_{\text{12}}{BMEET}_{i,t}+\beta_{\text{13}}{DUAL}_{i,t}+YEAR\;+\varepsilon_{i,t}, \end{array}$$ (8)

where MVC refers to the intellectual capital, PRIVATE is the privatization phenomenon. Following prior studies on intellectual capital and privatization [4,20,80,81], we include several factors as control variables such as assets growth (GROWTH) measured as the growth ratio of assets increase to total assets in prior year, firm age (FAGE) calculated as the number of years since the company’s establishment, while firm size (SIZE) is determined by taking the logarithm of total assets. Return on assets (ROA) measures profitability, and leverage (LEVERAGE) is assessed by the ratio of total liabilities to total assets. Tobin’s Q serves as an indicator of market valuation, and institutional ownership (INSTITUTION) is quantified as the proportion of shares held by institutional investors. Regional development (REGIOND) is a dummy coded 1 if a firm operate in more-developed region, board size (BOARD which is the number of directors on the firm directors board, board independence (BINDP) which is the ratio of independent directors on the board, board meeting (BMEET) is the number of meeting held by a firm yearly, the CEO duality (DUALITY) which is a dummy coded one if the CEO is the chairman at the same time. For more details see the Table 1.

**Table 1:** Variables descriptions.

Variable	Definition
MVC	Refers to the Intellectual Capital (IC) which calculated in three steps illustrated in section
PRIVATEPERCE	Reduction represents government withdrawal and is continuous variable indicating percentage of shares reduced by state owners by selling it to private investors, which varies 0–100 percent.
PRIVATDUM10P	A dummy variable coded one if the reduction percent is more than 10%, zero otherwise.
PRIVATDUM20P	A dummy variable coded one if the reduction percent is more than 20%, zero otherwise.
GROWTH	The ratio of assets growth for a firm in a year t
FAGE	The number of years since listing the firm
SIZE	The natural logarithm of total assets
ROA	Return on assets
LEVERAGE	The total liabilities to total assets
TOBINQ	TobinQ
INSTITUTION	The percentage of shareholders own by institutional investors
REGIOND	A dummy variable coded one if a firm operates in more-developed region, zero otherwise.
BOARD	The number of directors on the firm board directors
BINDP	The number of independent directors on the board
BMEET	The number of board meetings held a year
DUAL	A dummy coded one if the CEO is the chairman of the firm in the same time, zero otherwise
SOE	A dummy coded one if the firm controlled by the state or the government
INTERNATION	The ratio of foreign sales measured by foreign sales scaled by total sales.

## 4. Empirical results

### 4.1. Descriptive statistics

Table 2 presents the summary statistics of our regression variables. The average intellectual capital (MVC) is 3.717 with a standard deviation of 3.1, indicating substantial variation in intellectual capital levels across our sample firms. This value is broadly consistent with what reported by Xu & Wang [84], using Chinese firms, where the mean MVAIC typically ranges between 3.5 and 4.2. The mean privatization percentage (PRIVATEPERCE) is 22.461% with a standard deviation of 35.748%, suggesting considerable variation in state ownership reduction across the sample. This aligns with evidence from previous privatization studies in China and other emerging markets, which report average private ownership levels ranging from 20% to 30% following partial privatization [4]. Approximately 35% of firms experienced privatization of around 10% (PRIVATDUM10P), while 27.1% underwent privatization exceeding 20% (PRIVATDUM20P).

**Table 2:** Summary statistics.

	N	Mean	SD	p25	Median	p75	Skew	Kurt
MVC	13809	3.717	3.1	2.049	3.139	4.723	1.735	8.565
PRIVATEPERCE	13809	22.461	35.748	0	3.039	25.159	1.474	3.487
PRIVATDUM10P	13809	.35	.477	0	0	1	.631	1.398
PRIVATDUM20P	13809	.271	.445	0	0	1	1.029	2.059
GROWTH	13809	.343	10.267	.001	.079	.187	93.061	9656.777
FAGE	13809	13.136	7.579	7	13	19	.177	2.042
SIZE	13809	22.652	1.662	21.549	22.388	23.51	1.014	5.233
ROA	13809	.035	.156	.01	.031	.062	43.867	3089.995
LEVERAGE	13809	.49	.334	.311	.481	.645	17.304	587.764
TOBINQ	13809	1.947	1.909	1.092	1.471	2.165	15.716	531.166
INSTITUTION	13809	.456	.219	.303	.465	.622	−.133	2.307
BOARD	13809	8.917	1.89	8	9	9	1.022	5.888
BINDP	13809	3.287	.685	3	3	3	1.676	7.527
BMEET	13809	9.664	4.045	7	9	12	1.898	11.972
DUAL	13809	.202	.401	0	0	0	1.485	3.205
SOE	13809	.569	.495	0	1	1	−.279	1.078
REGIOND	13809	.771	.42	1	1	1	−1.289	2.66
INTERNATION	13809	.111	.239	0	.004	.13	32.774	1302.424

**Note**: This table shows a summary of the variables’ definitions.

Our sample’s corporate governance structure shows an average board size (BOARD) of 8.917 directors, with approximately 3.287 (36.9%) being independent directors (BINDP). Notably, 20.2% of firms have CEO duality (DUAL), indicating a moderate level of leadership concentration. The average firm age since listing (FAGE) is about 13 years, while the mean firm size (SIZE) is 22.652. The average return on assets (ROA) is 0.035, and the mean leverage ratio is 0.49, suggesting that firms finance about 49% of their assets with debt. Regarding ownership structure, 56.9% of the firms are state-owned enterprises (SOE), and 77.1% of the sample operate in more developed regions (REGIOND). The average level of internationalization (INTERNATION) is 11.1%, indicating a moderate degree of international market exposure.

### 4.2. Correlation

The correlation outcomes are reported in Table 3. The correlation coefficient between PRIVATEPERCE and MVC is significantly positive (correlation 0.022, p < 0.01), providing initial support for our hypothesis that privatization is positively associated with intellectual capital development. PRIVATEPERCE shows significant correlations with several firm characteristics. It is negatively correlated with firm age (FAGE) (correlation −0.216, p < 0.01) and firm size (SIZE) (correlation −0.175, p < 0.01), suggesting that comparatively newer and smaller firms tend to experience more privatization. The correlations between variables are generally consistent with expectations and provide preliminary insights into our hypotheses. Multicollinearity will not pose a substantial issue in the analysis, since all correlation coefficients between the independent variables fall below the threshold of 0.60. The highest correlation is between BOARD and BINDP (0.588, p < 0.01).

**Table 3:** Pairwise correlations.

Variables	(1)	(2)	(3)	(4)	(5)	(6)	(7)	(8)	(9)	(10)	(11)	(12)	(13)	(14)	(15)	(17)
MVC	1.000															
PRIVATEPERCE	0.022*	1.000														
PRIVATDUM10P	0.018*	0.790*	1.000													
PRIVATDUM20P	0.029*	0.888*	0.832*	1.000												
GROWTH	−0.014	0.013	0.014	0.014	1.000											
FAGE	−0.011	−0.216*	−0.201*	−0.218*	0.005	1.000										
SIZE	0.223*	−0.175*	−0.166*	−0.179*	−0.052*	0.204*	1.000									
ROA	0.133*	0.007	0.005	0.006	0.015	−0.031*	−0.009	1.000								
LEVERAGE	0.015	−0.083*	−0.070*	−0.087*	0.046*	0.150*	0.237*	−0.138*	1.000							
TOBINQ	−0.004	0.066*	0.076*	0.076*	−0.004	−0.043*	−0.302*	0.037*	−0.134*	1.000						
INSTITUTION	0.096*	−0.269*	−0.256*	−0.272*	−0.019	0.188*	0.412*	0.043*	0.058*	0.001	1.000					
BOARD	0.059*	−0.167*	−0.141*	−0.158*	−0.009	0.018	0.339*	−0.000	0.126*	−0.113*	0.204*	1.000				
BINDP	0.073*	−0.148*	−0.131*	−0.146*	−0.022*	0.035*	0.397*	−0.007	0.143*	−0.100*	0.207*	0.588*	1.000			
BMEET	0.066*	0.024*	0.039*	0.027*	−0.002	0.087*	0.232*	−0.040*	0.144*	−0.065*	0.038*	0.020	0.050*	1.000		
DUAL	0.005	0.175*	0.178*	0.181*	−0.007	−0.166*	−0.127*	0.012	−0.072*	0.066*	−0.169*	−0.169*	−0.126*	−0.016	1.000	
SOE	−0.046*	−0.457*	−0.465*	−0.484*	−0.008	0.245*	0.235*	−0.027*	0.115*	−0.095*	0.336*	0.195*	0.193*	0.013	−0.273*	1.000

*shows the significance level at 0.01.

### 4.3. Regression analysis

Table 4 highlights our main evidence examining the relationship between privatization and intellectual capital. Column (1) shows that PRIVATEPERCE has a positive and significant coefficient (0.002, p < 0.01), supporting our hypothesis that privatization enhances intellectual capital. Economically, a one-unit increase in PRIVATEPERCE is associated with a 0.2 increase in the MVC, holding other factors constant. This result is consistent with prior studies suggesting that private ownership promotes innovation and knowledge development [85,86].

**Table 4:** Privatization and intellectual capital.

	(1)	(2)	(3)
VARIABLES	MVC	MVC	MVC
PRIVATEPERCE	0.002***	–	–
	[2.78]	–	–
PRIVATDUM10P	–	0.135**	–
	–	[2.16]	–
PRIVATDUM20P	–	–	0.218***
	–	–	[3.17]
GROWTH	−0.002	−0.002	−0.002
	[-0.27]	[-0.28]	[-0.28]
FAGE	−0.033***	−0.034***	−0.033***
	[-5.03]	[-5.13]	[-5.02]
SIZE	0.489***	0.489***	0.489***
	[11.60]	[11.58]	[11.60]
ROA	2.226*	2.227*	2.229*
	[1.81]	[1.81]	[1.82]
LEVERAGE	−0.295*	−0.297*	−0.293*
	[-1.70]	[-1.71]	[-1.69]
TOBINQ	0.092***	0.092***	0.092***
	[2.83]	[2.82]	[2.80]
INSTITUTION	0.101	0.087	0.106
	[0.55]	[0.47]	[0.57]
BOARD	−0.061**	−0.063**	−0.062**
	[-2.05]	[-2.11]	[-2.07]
BINDP	−0.048	−0.047	−0.047
	[-0.55]	[-0.54]	[-0.54]
BMEET	−0.006	−0.006	−0.006
	[-0.67]	[-0.67]	[-0.69]
DUAL	0.088	0.088	0.087
	[0.95]	[0.95]	[0.94]
SOE	−0.462***	−0.474***	−0.447***
	[-4.40]	[-4.50]	[-4.24]
Constant	−5.269***	−5.229***	−5.279***
	[-5.09]	[-5.06]	[-5.11]
Industry & Year	Yes	Yes	Yes
Observations	13,785	13,785	13,785
Adj.R2	0.32	0.32	0.32

**Note**: This table reports the results of the relationship between privatization and intellectual capital. Robust *t*-statistics (clustered at the firm level) are reported in parentheses. *, ** and *** denote a two-tailed *p*-value of less than 0.10, 0.05 and 0.01, respectively.

Columns (2) and (3) use alternative measures of privatization, i.e., PRIVATDUM10P and PRIVATDUM20P and show consistent results. The coefficient for PRIVATDUM10P is positive and significant (0.135, p < 0.05), indicating that firms experiencing more than 10% privatization demonstrate higher intellectual capital. Similarly, PRIVATDUM20P shows a stronger positive effect (0.218, p < 0.01), suggesting that more substantial privatization leads to greater intellectual capital development. These findings support the argument that private ownership creates stronger incentives for intellectual capital investment [87].

Regarding control variables, firm size (SIZE) shows a consistently positive and significant relationship with intellectual capital across all specifications (coefficient = 0.489, p < 0.01), indicating that larger firms tend to have higher intellectual capital. Conversely, firm age (FAGE) shows a negative relationship (coefficient = −0.033, p < 0.01), suggesting that comparatively newer firms tend to be more dynamic in developing intellectual capital. The negative and significant coefficient for SOE status (coefficient = −0.462, p < 0.01) further supports the benefits of private ownership for intellectual capital development.

### 4.4. The moderating effect of internationalization

Table 5 presents the results examining how internationalization moderates the relationship between privatization and intellectual capital. The baseline effect of PRIVATEPERCE remains positive and significant (0.003, p < 0.01), consistent with our main findings. However, the interaction term PRIVATEPERCE*INTERNATION is negative and significant (−0.005, p < 0.10), suggesting that internationalization weakens the positive effect of privatization on intellectual capital. This moderating effect is further confirmed using our alternative privatization measures. The interaction term PRIVATDUM10P* INTERNATION (−0.371, p < 0.10) and PRIVATDUM20P*INTERNATION (−0.496, p < 0.05) are both negative and significant. These findings suggest that firms with higher international exposure may experience reduced benefits from privatization in terms of intellectual capital development.

**Table 5:** The results of the moderating effect of internationalization.

	(1)	(2)	(3)
VARIABLES	MVC	MVC	MVC
PRIVATEPERCE	0.003***	–	–
	[3.34]	–	–
INTERNATION	−0.941***	−0.953***	−0.945***
	[-7.26]	[-7.58]	[-7.44]
PRIVATEPERCE* INTERNATION	−0.005*	–	–
	[-1.80]	–	–
PRIVATDUM10P	–	0.177**	–
	–	[2.51]	–
PRIVATDUM10P* INTERNATION	–	−0.371*	–
	–	[-1.71]	–
PRIVATDUM20P	–	–	0.271***
	–	–	[3.46]
PRIVATDUM20P* INTERNATION	–	–	−0.496**
	–	–	[-2.00]
GROWTH	−0.002	−0.002	−0.002
	[-0.26]	[-0.33]	[-0.32]
FAGE	−0.034***	−0.048***	−0.048***
	[-5.15]	[-7.53]	[-7.44]
SIZE	0.503***	0.412***	0.412***
	[11.98]	[10.28]	[10.29]
ROA	2.219*	2.364*	2.366*
	[1.81]	[1.82]	[1.83]
LEVERAGE	−0.298*	−0.153	−0.149
	[-1.74]	[-1.09]	[-1.06]
TOBINQ	0.090***	0.080***	0.080***
	[2.78]	[2.63]	[2.60]
INSTITUTION	0.127	0.202	0.220
	[0.69]	[1.08]	[1.17]
BOARD	−0.061**	−0.036	−0.035
	[-2.05]	[-1.21]	[-1.18]
BINDP	−0.059	−0.035	−0.035
	[-0.67]	[-0.41]	[-0.41]
BMEET	−0.007	−0.002	−0.002
	[-0.75]	[-0.19]	[-0.21]
DUAL	0.112	0.069	0.069
	[1.21]	[0.75]	[0.75]
SOE	−0.491***	−0.410***	−0.384***
	[-4.70]	[-3.92]	[-3.67]
Constant	−5.495***	−4.660***	−4.696***
	[-5.35]	[-4.58]	[-4.63]
Industry & Year	Yes	Yes	Yes
Observations	13,785	13,785	13,785
Adj.R2	0.32	0.31	0.31

**Note**: This table reports the results of the moderating effect of internationalization on the relationship between privatization and intellectual capital. Robust *t*-statistics (clustered at the firm level) are reported in parentheses. *, ** and *** denote a two-tailed *p*-value of less than 0.10, 0.05 and 0.01, respectively.

### 4.5. The moderating effect of regional development

Table 6 shows how regional development influences the privatization-intellectual capital relationship. The interaction term PRIVATEPERCEREGIOND is positive and significant (0.013, p < 0.10), indicating that the positive effect of privatization on intellectual capital is stronger in more developed regions. This finding is consistent across alternative measures of privatization, with both PRIVATDUM10P*REGIOND (0.182, p < 0.10) and PRIVATDUM20P*REGIOND (0.139, p < 0.10) showing positive and significant coefficients.

**Table 6:** The results of the moderating effect of regional development.

	(1)	(2)	(3)
VARIABLES	MVC	MVC	MVC
PRIVATEPERCE	0.005**	–	–
	[2.53]	–	–
REGIOND	0.053	0.056	0.027
	[0.49]	[0.51]	[0.25]
PRIVATEPERCE* REGIOND	0.013*	–	–
	[1.72]	–	–
PRIVATDUM10P	–	0.276**	–
	–	[2.13]	–
PRIVATDUM10P* REGIOND	–	0.182*	–
	–	[1.79]	–
PRIVATDUM20P	–	–	0.329**
	–	–	[2.27]
PRIVATDUM20P* REGIOND	–	–	0.139*
	–	–	[1.89]
GROWTH	−0.002	−0.002	−0.002
	[-0.27]	[-0.28]	[-0.28]
FAGE	−0.034***	−0.034***	−0.033***
	[-5.05]	[-5.15]	[-5.04]
SIZE	0.489***	0.488***	0.489***
	[11.59]	[11.56]	[11.59]
ROA	2.228*	2.228*	2.229*
	[1.81]	[1.81]	[1.82]
LEVERAGE	−0.293*	−0.296*	−0.292*
	[-1.69]	[-1.70]	[-1.68]
TOBINQ	0.092***	0.092***	0.092***
	[2.82]	[2.81]	[2.79]
INSTITUTION	0.100	0.085	0.104
	[0.54]	[0.46]	[0.56]
BOARD	−0.062**	−0.063**	−0.062**
	[-2.07]	[-2.12]	[-2.08]
BINDP	−0.047	−0.046	−0.047
	[-0.54]	[-0.53]	[-0.54]
BMEET	−0.006	−0.006	−0.006
	[-0.72]	[-0.69]	[-0.71]
DUAL	0.090	0.089	0.088
	[0.97]	[0.96]	[0.95]
SOE	−0.460***	−0.474***	−0.446***
	[-4.37]	[-4.50]	[-4.23]
Constant	−5.285***	−5.245***	−5.283***
	[-5.12]	[-5.07]	[-5.12]
Industry & Year	Yes	Yes	Yes
Observations	13,785	13,785	13,785
Adj.R2	0.32	0.32	0.32

**Note**: This table reports the results of the moderating effect of regional development level on the relationship between privatization and intellectual capital. Robust *t*-statistics (clustered at the firm level) are reported in parentheses. *, ** and *** denote a two-tailed *p*-value of less than 0.10, 0.05 and 0.01, respectively. Variable definitions are provided in the appendix.

These results align with institutional theory and suggest that more developed regions provide better institutional infrastructure and support systems that enhance the effectiveness of privatization in building intellectual capital. This finding is particularly relevant for emerging economies where regional development disparities can significantly influence firm outcomes [15].

### 4.6. The generalized method of moments (GMM) test

As we find a positive relationship between privatization and intellectual capital, this claim may be subject to reverse causality, which could introduce potential bias. The dynamic nature of intellectual capital development may create endogeneity issue. The GMM technique itself effectively addresses endogeneity problems by leveraging internal instruments, such as lagged values of the independent variables, which are correlated with the endogenous explanatory variables but remain uncorrelated with the error term. By exploiting such lagged values allows GMM to capture variations in the MVC that are unaffected by current privatization activities, thereby mitigating bias arising from potential reverse causality. Table 7 presents the results of our GMM estimation. The lagged dependent variable (L.MVC) is positive and significant across all specifications (0.363 to 0.733, p < 0.01), indicating strong persistence in intellectual capital levels.

**Table 7:** Results of the GMM model.

	(1)	(4)	(3)
VARIABLES	MVC	MVC	MVC
L.MVC	0.363***	0.723***	0.733***
	[27.45]	[121.51]	[121.04]
PRIVATEPERCE	0.001***	0.002***	0.003***
	[2.77]	[5.38]	[4.27]
INTERNATION		−0.376***	
		[-3.96]	
PRIVATEPERCE* INTERNATION		−0.004**	
		[-2.38]	
REGIOND			0.076*
			[1.70]
PRIVATEPERCE* REGIOND			0.002***
			[3.03]
GROWTH	0.006	0.005***	0.005***
	[1.01]	[13.04]	[8.72]
FAGE	−0.045***	0.004***	0.005***
	[-4.91]	[2.79]	[3.14]
SIZE	0.091**	0.019**	0.009
	[2.14]	[2.01]	[0.91]
ROA	10.388***	7.560***	7.350***
	[28.87]	[38.20]	[36.43]
LEVERAGE	0.953***	0.795***	0.760***
	[6.51]	[14.12]	[13.56]
TOBINQ	0.011	0.002	0.001
	[1.10]	[0.40]	[0.14]
INSTITUTION	−0.092	0.000	−0.005
	[-1.11]	[0.01]	[-0.12]
BOARD	−0.022*	−0.005	−0.010
	[-1.70]	[-0.67]	[-1.42]
BINDP	−0.016	−0.040**	−0.015
	[-0.46]	[-2.00]	[-0.74]
BMEET	−0.014***	0.012***	0.012***
	[-3.91]	[5.82]	[5.41]
DUAL	0.107***	0.050**	0.024
	[2.82]	[2.50]	[1.20]
SOE	−0.260***	−0.347***	−0.148***
	[-3.37]	[-3.50]	[-3.23]
Constant	0.000	−0.234	−0.087
	[.]	[-1.21]	[-0.44]
Observations	12,672	12,672	12,672
AR(1)	z = −6.00, P = 0.000	z = −6.10, P = 0.000	z = −6.03, P = 0.000
AR(1)	z = 0.47, P = 0.640	z = 0.55, P = 0.621	z = 0.53, P = 0.590
Sargan test	chi2 = 1040.70, P = 0.000	chi2 = 1122.51, P = 0.000	chi2 = 1160.94, P = 0.000
Hansen test	chi2 = 572.51, P = 0.264	chi2 = 617.71, P = 0.330	chi2 = 672.10, P = 0.285

**Note**: This table reports the results of the GMM model for the relationship between privatization and intellectual capital. Robust *t*-statistics (clustered at the firm level) are reported in parentheses. *, ** and *** denote a two-tailed *p*-value of less than 0.10, 0.05 and 0.01, respectively. Variable definitions are provided in the appendix.

The findings of GMM offer robust support for our main findings: The direct effect of privatization (PRIVATEPERCE) remains positive and significant (0.001 to 0.003, p < 0.01) across all specifications. The moderating effect of internationalization (PRIVATEPERCE*INTERNATION) is confirmed to be negative and significant (−0.004, p < 0.05). Also, the regional development interaction (PRIVATEPERCE*REGIOND) remains positive and significant (0.002, p < 0.01).

The validity of our GMM estimation is supported by the significant AR(1) but insignificant AR(2) test results, indicating no second-order serial correlation. The significant Sargan test, and insignificant Hansen test results, supporting the validity of our instruments. These dynamic panel results provide strong evidence that our findings are robust to potential endogeneity concerns and the dynamic nature of intellectual capital development.

### 4.7. The propensity score matching (PSM) approach

Our results may suffer from the endogeneity arising from observable factors rather than from unobservable. Addressing this concern, we utilize the PSM model. The results of the propensity score matching (PSM) analysis, presented in Table 8, offer valuable insights into the relationship between privatization and intellectual capital. **Panel A** shows the success of the matching process, with no statistically significant differences between the treated (privatized) and control (non-privatized) firms across a range of firm characteristics. In **Panel B**, Model (3) displays the baseline effect of the privatization measure (PRIVATEPERCE) on intellectual capital (MVC). The coefficient of PRIVATEPERCE is positive and highly significant coefficient (0.002, p < 0.01) suggests that greater levels of privatization are associated with enhanced intellectual capital development. This evidence supports this idea that private ownership creates stronger motivations for companies to capitalize on knowledge-based assets and innovation.

**Table 8:** Results of the PSM: (Panel A) Results of matching process.

Variable	Treated	Controls	Difference	S.E.	T-stat
GROWTH	0.538	0.353	0.186	0.248	0.750
FAGE	11.059	11.256	−0.196	0.171	−1.150
SIZE	22.277	22.292	−0.016	0.038	−0.410
ROA	0.036	0.034	0.002	0.004	0.480
LEVERAGE	0.458	0.468	−0.010	0.008	−1.280
TOBINQ	2.145	2.116	0.029	0.045	0.640
INSTITUTION	0.380	0.382	−0.002	0.005	−0.380
BOARD	8.556	8.625	−0.068	0.043	−1.580
BINDP	3.165	3.179	−0.014	0.016	−0.890
BMEET	9.887	9.873	0.014	0.093	0.150
DUAL	0.300	0.305	−0.005	0.009	−0.570
SOE	0.256	0.255	0.001	0.010	0.070
**Panel B: The results of PSM**					
			**(1)**	**(2)**	**(3)**
**VARIABLES**			**MVC**	**MVC**	**MVC**
PRIVATEPERCE			0.002***	0.003***	0.003*
			[2.68]	[3.05]	[1.83]
INTERNATION			–	−1.090***	–
			–	[-6.10]	–
PRIVATEPERCE* INTERNATION			–	−0.004*	–
			–	[-1.85]	–
REGIOND			–	–	0.075
			–	–	[0.73]
PRIVATEPERCE* REGIOND			–	–	0.001**
			–	–	[2.42]
GROWTH			0.001	0.001	0.001
			[0.12]	[0.20]	[0.12]
FAGE			−0.037***	−0.040***	−0.037***
			[-7.34]	[-7.83]	[-7.34]
SIZE			0.496***	0.511***	0.496***
			[15.03]	[15.45]	[15.03]
ROA			1.874**	1.875*	1.874**
			[1.96]	[1.96]	[1.96]
LEVERAGE			−0.322***	−0.323***	−0.321***
			[-2.82]	[-2.86]	[-2.82]
TOBINQ			0.088***	0.085***	0.089***
			[3.87]	[3.80]	[3.87]
INSTITUTION			0.179	0.219	0.179
			[1.13]	[1.40]	[1.13]
BOARD			−0.036	−0.031	−0.037
			[-1.54]	[-1.30]	[-1.55]
BINDP			−0.216***	−0.229***	−0.215***
			[-3.08]	[-3.27]	[-3.07]
BMEET			−0.004	−0.005	−0.004
			[-0.53]	[-0.65]	[-0.55]
DUAL			−0.029	−0.011	−0.029
			[-0.43]	[-0.16]	[-0.43]
SOE			−0.447***	−0.478***	−0.447***
			[-6.33]	[-6.78]	[-6.32]
Constant			−4.845***	−5.173***	−4.858***
			[-5.97]	[-6.36]	[-5.98]
Observations			4,818	4,818	4,818
Adj.R2			0.29	0.30	0.29

**Note**: This table reports the results of the PSM model for the relationship between privatization and intellectual capital. Robust *t*-statistics (clustered at the firm level) are reported in parentheses. *, ** and *** denote a two-tailed *p*-value of less than 0.10, 0.05 and 0.01, respectively. Variable definitions are provided in the appendix.

Model (2) shows estimates for the moderating role of internationalization (INTERNATION). The negative and significant interaction term PRIVATEPERCE*INTERNATION (−0.004, p < 0.10) indicates that the positive effect of privatization on intellectual capital is weakened for firms with more international exposure. Finally, Model (5) investigates the moderating influence of regional development (REGIOND). The positive and significant coefficient on the interaction term PRIVATEPERCE*REGIOND (0.001, p < 0.05) suggests that the benefits of privatization for intellectual capital are amplified in more developed regions. In sum, these finding suggest that our findings are robust for endogeneity problems.

## 5. Discussion

Our analysis shows a positive relationship between the privatization and intellectual capital. Our study claims that privatization can improve intellectual capital by bringing enhanced managerial autonomy, providing leaders opportunities to make strategic choices without limitations which typically imposed by state holding. As to private companies, managers are likely have greater incentives to improve efficiency, innovate, and invest in employee development, all of which contribute to intellectual capital. In private ownership structures, managerial rewards are frequently tied to their performance, which encourages actions that enhance the firm’s long-term value, including investment in knowledge assets [88]. Moreover, these firms can have more freedom to allocate resources toward intellectual capital development, such as R&D, training, and technology adoption. While SOEs often face restrictions due to the social and political objectives that limit their capacity for such investments. We believe that, unlike SOEs, shifting to private ownership can foster a culture that values intellectual capital.

Extending this debate, our study demonstrates that internationalization negatively moderates the relationship between privatization and intellectual capital. Particularly, internationalized firms often encounter complexities related to diverse regulatory environments, cultural differences, and operational hurdles in foreign markets. These challenges can limit the ability of privatized firms to fully leverage the autonomy and efficiency gains associated with privatization for intellectual capital growth. The “liability of foreignness” perspective suggests that such complexities may constrain firms’ ability to transfer and integrate intellectual assets across borders, diminishing the benefits that privatization typically offers. Further, internationalized firms may need to allocate more resources toward managing foreign operations, which could detract from investments in intellectual capital development. In contrast, firms operating primarily in domestic markets can channel resources more directly into developing intellectual assets like knowledge, skills, and innovative capacities. This aligns with the finding that the positive effect of privatization on intellectual capital is stronger in less internationalized firms.

Finally, the findings reveal that the regional development positively moderates the positive association between privatization and intellectual capital. In light of institutional theory [89], the effectiveness of organizational practices, including privatization, is influenced by the surrounding institutional environment. In more developed regions, stronger institutional support, better infrastructure, and access to skilled labor create a conducive environment for firms to leverage the benefits of privatization effectively. These regions often provide superior access to capital markets, educational resources, and legal frameworks [90], all of which are essential for developing intellectual capital. In less developed regions, however, the absence of such institutional support and infrastructure may constrain the potential benefits of privatization. Firms in these areas might lack access to key resources, limiting their ability to fully invest in intellectual capital, such as research and development (R&D), employee training, and innovative practices. This limitation may reduce the overall impact of privatization on knowledge and innovation.

## 6. Implications

This study also has a number of implications, particularly for policymakers and corporate decision makers who are interested in enhancing intellectual capital development. For instance, the present study educates the policy makers regarding the possible consequences of partial privatization as a mechanism to promote intellectual capital development in privatized companies. By having a mix of private and public and private owners in firms, they may leverage private shareholders’ focus on operational efficiency and accountability. This notion supports the public choice theory that introducing private owners introduce an additional layer of monitoring, ultimately weaken the influence of purely political objectives [26,27]. In this context, partial privatization may assist corporations to allocate resources more efficiently, eventually fostering better utilization of intellectual capital for sustained growth and competitive advantage.

In view of agency standpoint, our study supports the principles of agency theory, highlighting the advantages of privatization in reducing inefficiencies caused by the principal-agent problem [39–41]. The increasing participation of private shareholders shifts the focus from political agendas to corporate strategic agendas centered on value creation. Particularly, such kind of shift is vital to promote innovation and productivity within partially privatized firms, allowing them to arrange and manage intellectual capital more effectively. In a case of privatization, firms will be freed from excessive government involvement, they can be more likely to invest in human capital, research and development, and other intangible assets that drive long-term value creation. This redirection of focus from regulatory compliance to strategic initiatives is crucial for firms operating in competitive environments.

Along this, the present study suggests that the privatization of government ownership could serve as a policy recommendation for countries aiming to balance state control with private sector efficiencies. By having partial hold of government while introducing private stakeholders, strategists may achieve a dual objective in a firm: ensuring public interest protection while also fostering a performance-driven culture within enterprises. This hybrid approach can help governments optimize their control over strategic industries without compromising on productivity and innovation, thus ensuring sustainable economic growth.

Instead of providing direct effects of privatization, our study further provides implications on the roles of corporate internationalization and regional development. The negative moderating effect of internationalization implies that as companies expand their operations globally, the consequences of partial privatization on intellectual capital development may not be satisfactory. This could be due to the complexities and risks associated with managing diverse operations across different countries and cultures, which may dilute the focus on efficiency and innovation. Regulatory bodies and corporate leaders should recognize that while internationalization can open new markets, it may also introduce additional layers of complexity that could hinder the efficient utilization of intellectual capital.

Besides, this study suggests that regional development highlights the critical role of the local business environment in enhancing the outcomes of partial privatization. Privatized companies located in regions with advanced infrastructure, financial and technological development, and advanced policies are better positioned to capitalize on the benefits of partial privatization. Firms operating in less developed regions often contend with weaker institutional environments, which can diminish their responsiveness to broader economic growth and impede the process of capital building. This implies that policymakers should focus on strengthening regional development initiatives to provide an enabling environment that enhances firms’ ability to leverage intellectual capital. By fostering regional clusters of innovation and productivity, governments can ensure that partially privatized firms achieve their full potential, thereby contributing to overall economic growth and development.

## 7. Conclusion

Prior studies have predominantly concentrated on examining the economic impact of privatization efforts at a macro level, or on assessing financial performance at a micro level. However, when it comes to firm-level analysis, our knowledge of how secondary privatization influences intellectual capital efficiency remains in its early stages. Utilizing a sample of Chinese firms over the period from 2010 to 2023, the present study provides evidence that partial privatization can positively impact intellectual capital, ultimately supporting value creation within firms. However, further examinations submit that the benefits of this relationship are not uniformly realized; firm internationalization emerges as a factor that weakens the positive effect of partial privatization on intellectual capital. The complexity and diversity of managing operations in international markets appear to shift the focus away from optimizing capital efficiency, as companies face additional challenges related to regulatory compliance, cultural differences, and operational intricacies. This suggests that firms must strategically manage their international operations to sustain the gains achieved through partial privatization. Instead, the positive moderating impact of regional development emphasizes the importance of a robust local business ecosystem. Companies operating in in developed regions are better equipped to leverage the benefits of partial privatization to enhance their intellectual capital. By focusing on China—a country with distinctive ownership structures and regulatory frameworks—our findings provide critical insights into how partial privatization can be strategically utilized in emerging markets to foster sustainable growth. The study also emphasizes the necessity for stronger governance frameworks and regulatory mechanisms to maximize the advantages of privatization initiatives. Ultimately, our work not only enriches the understanding of the interplay between privatization and intellectual capital but also offers actionable recommendations for firms and policymakers aiming to create a more efficient and transparent business environment.

This study also bears some limitations. For instance, the current research focuses Chinese context, hence policies and institutional regulations of China differ from any other countries, so its general results may hardly be applicable to other emerging markets. Moreover, while this research covers the institutional variables of firm internationalization and regional development, other important institutional variations are omitted, such as subnational institutional differences which depict larger institutional varieties within China. Future research should therefore explore these institutional differences with a view to deepening the understanding of how privatization functions under different regulatory settings. It would also be useful if increasingly complex models were included, such as any mediating or moderating influences of structures of corporate governance or regulatory enforcement, which would provide further detail on the mechanisms underlying the outcomes of privatization.

## Supporting information

S1 File Dataset. (XLSX)
